# Altered Chromosomal Positioning, Compaction, and Gene Expression with a Lamin A/C Gene Mutation

**DOI:** 10.1371/journal.pone.0014342

**Published:** 2010-12-14

**Authors:** Stephanie K. Mewborn, Megan J. Puckelwartz, Fida Abuisneineh, John P. Fahrenbach, Yuan Zhang, Heather MacLeod, Lisa Dellefave, Peter Pytel, Sara Selig, Christine M. Labno, Karen Reddy, Harinder Singh, Elizabeth McNally

**Affiliations:** 1 Department of Medicine, The University of Chicago, Chicago, Illinois, United States of America; 2 Department of Human Genetics, The University of Chicago, Chicago, Illinois, United States of America; 3 Department of Pathology, The University of Chicago, Chicago, Illinois, United States of America; 4 Molecular Medicine Laboratory, Rambam Health Care Campus and Rappaport Faculty of Medicine and Research Institute, Technion - Israel Institute of Technology, Haifa, Israel; 5 Howard Hughes Medical Institute and Department of Molecular Genetics and Cell Biology, The University of Chicago, Chicago, Illinois, United States of America; Duke University, United States of America

## Abstract

**Background:**

Lamins A and C, encoded by the *LMNA* gene, are filamentous proteins that form the core scaffold of the nuclear lamina. Dominant *LMNA* gene mutations cause multiple human diseases including cardiac and skeletal myopathies. The nuclear lamina is thought to regulate gene expression by its direct interaction with chromatin. *LMNA* gene mutations may mediate disease by disrupting normal gene expression.

**Methods/Findings:**

To investigate the hypothesis that mutant lamin A/C changes the lamina's ability to interact with chromatin, we studied gene misexpression resulting from the cardiomyopathic *LMNA* E161K mutation and correlated this with changes in chromosome positioning. We identified clusters of misexpressed genes and examined the nuclear positioning of two such genomic clusters, each harboring genes relevant to striated muscle disease including *LMO7* and *MBNL2*. Both gene clusters were found to be more centrally positioned in *LMNA*-mutant nuclei. Additionally, these loci were less compacted. In *LMNA* mutant heart and fibroblasts, we found that chromosome 13 had a disproportionately high fraction of misexpressed genes. Using three-dimensional fluorescence in situ hybridization we found that the entire territory of chromosome 13 was displaced towards the center of the nucleus in *LMNA* mutant fibroblasts. Additional cardiomyopathic *LMNA* gene mutations were also shown to have abnormal positioning of chromosome 13, although in the opposite direction.

**Conclusions:**

These data support a model in which *LMNA* mutations perturb the intranuclear positioning and compaction of chromosomal domains and provide a mechanism by which gene expression may be altered.

## Introduction

The nuclear membrane is composed of a two distinct lipid bilayers, and the inner nuclear membrane is composed of nucleoplasmic and transmembrane proteins. The lamins are intermediate filament proteins that form a scaffold intimately linked to the inner nuclear membrane where they provide shape and mechanical stability to the nucleus. This inner nuclear lamina is involved in multiple distinct cellular processes, including nuclear assembly, apoptosis, signal transduction, transport, and chromosome segregation [1], [2], [3], [4]. The lamins contain a central α-helical coiled-coil rod domain that mediates the formation of the higher ordered structures that comprise the lamina.

Lamins B1 and B2 are more highly expressed in mitotically active cells, and lamins A and C are expressed in post-mitotic cells [5], [6]. Lamins A and C are produced from the same gene and are identical for the first 566 amino acids. Over 300 different mutations associated with the *LMNA* gene have been described in a diverse list of overlapping phenotypes. Some of these phenotypes, especially those of striated muscle, represent a spectrum of disease [7]. Known as laminopathies, these disorders include cardiac and skeletal myopathies, lipodystrophies, neuropathies, and premature aging syndromes. Point mutations, frameshift mutations, deletions, and nonsense mutations all contribute to the pathogenesis of the laminopathies, and most mutations are dominant. The mechanism by which *LMNA* mutations alter the function of the nuclear membrane and cause disease is still unclear.

The nuclear lamina directly binds several key nuclear membrane proteins, including the SUN proteins, nesprins and emerin, as well as transcription regulators [5], [8], [9], [10], [11]. The role of the nuclear lamina in regulating gene expression has been increasingly appreciated. Relocalization to the nuclear membrane has been shown to be sufficient to repress gene expression [12], [13], [14], [15]. There are several points of contact between the nuclear lamina and chromatin to mediate gene expression. Barrier to autointegration factor (BAF) bridges DNA and A-type lamins [16]. The lamins also associate with Lamin-associated Protein 2 (LAP2), which binds to BAF, thereby establishing an additional connection between the nuclear lamina and chromatin [5]. Recent studies suggest that the A-type and B-type lamins form microdomains in the nucleus, with each type of lamin interacting with a different chromatin state [17]. These microdomains may serve as anchorage sites for heterochromatin; disruption of the lamina may alter the heterochromatin and euchromatin organization within the nucleus [17]. Chromatin organization may involve the direct binding of inner nuclear membrane proteins and transcriptional machinery, such as the lim domain only 7 (LMO7) protein, a transcriptional activator that shuttles back and forth between the nucleus and cytoplasm and binds emerin at the nuclear membrane [10]. These observations of the nuclear lamina's location and associated protein complexes support the lamina's role in transcriptional regulation and heterochromatin organization [7], [18].

The mechanism by which *LMNA* gene mutations lead to disease may be multifactorial but likely includes inducing aberrant gene expression. Mislocalization of chromosomes has been noted in *LMNA* mutant cells [19], [20]. We sought to extend these findings by querying whether an *LMNA* mutation was associated with a correlation between chromosome malpositioning and aberrant gene expression. We profiled gene expression in an *LMNA* mutant heart available after transplant from an individual with dilated cardiomyopathy (DCM), a condition characterized by the enlargement of the left ventricle and reduced cardiac function. We found that chromosome 13 had an overrepresentation of misexpressed genes and, correspondingly, the entire chromosome 13 territory was displaced towards the nuclear center. We identified several genomic clusters on chromosome 13 containing misexpressed genes relevant to muscle disease. By three dimensional fluorescence in situ hybridization (FISH) analyses, we show that both clusters were displaced towards the center of the nucleus in *LMNA*-mutant fibroblast cells. These data support a role for the nuclear lamina in the scaffolding of chromatin and the regulation of gene expression. Moreover, these data reconfirm that mutations in lamin A/C may alter the nuclear positioning of chromosomes.

## Results

### *LMNA* E161K mutant cardiomyopathic hearts have altered gene expression

**Figure 1:**
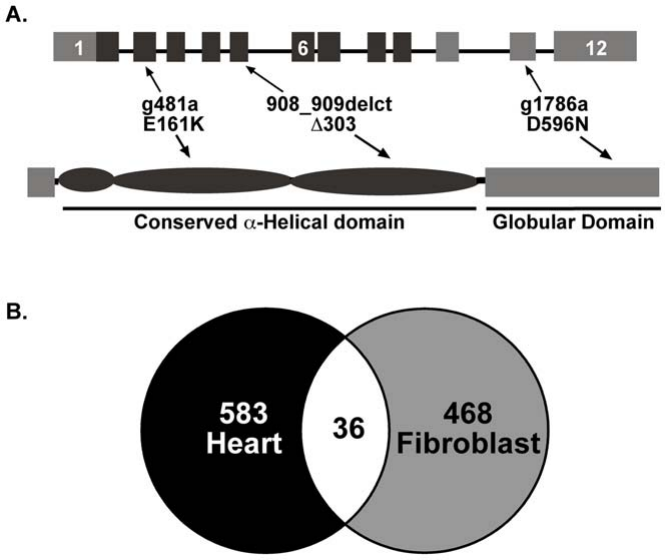
*LMNA* mutations cause dilated cardiomyopathy and disrupt gene expression. (A) Genomic (top) and protein structure (bottom) of the *LMNA* gene. The positions of the *LMNA* mutations analyzed in this study are shown. The E161K mutation was characterized in depth because cardiac material was available. Fibroblasts were used from all three *LMNA* mutants. All three mutations associate with cardiomyopathy with variable muscle disease. (B) Venn diagram of number of misexpressed genes in *LMNA* E161K heart (black) and fibroblasts (gray), shared genes are in white.

*LMNA* E161K was identified in an individual with familial dilated cardiomyopathy (Figure 1A). This individual was found to have a severely dilated heart with reduced function. As with other *LMNA* mutations, cardiac conduction system disease was present with atrial fibrillation accompanied by a slow ventricular rate [21], [22], [23], [24]. This mutation, *LMNA* E161K, has been previously reported in familial cardiomyopathy where it was shown to segregate with dilated cardiomyopathy and atrial fibrillation, and was not found in normal controls [25], [26]. The individual carrying this mutation underwent cardiac transplantation.

The available explanted heart was available for profiling gene expression. As a control, gene expression changes were profiled from an adult, male heart that also presented as end-stage dilated cardiomyopathy but was *LMNA* normal. The regions of the hearts selected for RNA preparation were derived from similar regions of the left ventricle that were devoid of grossly visible fibrosis. We compared the gene expression between the two end-stage cardiomyopathic hearts reasoning that the differences in gene expression were more likely to reflect the *LMNA* mutation state. We identified 674 probesets on the Affymetrix HGU133 plus 2.0 chip that were misregulated in the *LMNA* E161K mutant heart. These 674 probesets correspond to 583 unique genes differentially expressed between the end-stage *LMNA* E161K mutant and *LMNA* normal hearts, with 241 genes over-expressed in the *LMNA* E161K mutant heart and 342 genes under-expressed in the *LMNA* E161K mutant heart. With only a single *LMNA* mutant heart available for microarray analysis, common data analysis tools were inappropriate since these approaches would have a high incidence of false positives. To counter this effect, we used the Ranked Products algorithm to generate our list of misregulated genes.

Due to the limited availability of patient heart tissue, we also interrogated gene expression changes from *LMNA* E161K mutant fibroblasts compared to a control fibroblast line from a *LMNA* normal individual. Using the same Affymetrix array used for the heart analysis, we identified 500 probesets corresponding to 468 unique genes that were misregulated in the *LMNA* E161K mutant fibroblast line compared to the *LMNA* normal fibroblasts. Of these genes, 215 were over-expressed and 253 were under-expressed in the *LMNA* E161K mutant fibroblasts compared to the *LMNA* normal fibroblasts. We similarly used the Ranked Products algorithm to generate the gene list in the same manner as for the heart data. The gene lists for the *LMNA* E161K heart and fibroblasts were compared and we identified 36 unique genes that were misregulated in both the *LMNA* E161K heart and fibroblasts (Figure 1B). Table 1 provides the microarray data for genes misregulated in both heart and fibroblasts.

**Table 1:** Genes misexpressed in both LMNA mutant heart and fibroblasts.

Heart Microarray Fold Change	Fibroblast MicroarrayFold Change	Symbol	Gene Name	GenBank ID	Chromosome #
−7.4	2.4	ATP1B1	ATPase, Na+/K+ transporting, beta 1 polypeptide	NG_023230.1	1
2.6	2.8	BRP44	brain protein 44	NM_001143674	1
−10.8	−2	LEPROT	leptin receptor overlapping transcript	NM_017526.3	1
−8.9	3.3	PPAP2B	phosphatidic acid phosphatase type 2B	NM_003713.3	1
4.4	−4.3	PRELP	proline/arginine-rich end leucine-rich repeat protein	NM_002725.3	1
−4.5	−2.3	TXNIP	thioredoxin interacting protein	NM_006472.3	1
−2.4	−1.54	ZNF281	zinc finger protein 281	NM_012482.3	1
23.9	1.9	CYP1B1	cytochrome P450, family 1, subfamily B, polypeptide 1	NG_008386.1	2
−2	2	ID2	inhibitor of DNA binding 2, dominant negative helix-loop-helix protein	NM_002166.4	2
2.6	11.1	PCOLCE2	procollagen C-endopeptidase enhancer 2	NM_013363.3	3
−6.5	−1.7	ZBTB38	zinc finger and BTB domain containing 38	NG_021426.1	3
3.3	2.4	C5orf23	chromosome 5 open reading frame 23	NM_024563.3	5
2.5	1.8	DUSP1	dual specificity phosphatase 1	NM_004417.3	5
4	−38.3	EDIL3	EGF-like repeats and discoidin I-like domains 3	NM_005711.3	5
3.4	−28.7	PDCD6	programmed cell death 6	NM_013232.3	5
−8.5	−1.9	RHOBTB3	Rho-related BTB domain containing 3	NM_014899.3	5
8.3	−3.5	COL12A1	collagen, type XII, alpha 1	NM_004370.5	6
−2.1	−1.9	RUNX1T1	runt-related transcription factor 1; translocated to, 1 (cyclin D-related)	NG_023272.1	8
7.7	2.1	KLF4	Kruppel-like factor 4 (gut)	NM_004235.4	9
−3.8	−1.9	UGCG	UDP-glucose ceramide glucosyltransferase	NM_003358.1	9
−2.2	−1.9	C11orf54	chromosome 11 open reading frame 54	NM_014039.2	11
10.1	−10.8	LOC387758/FIBIN	fin bud initiation factor homolog (zebrafish)	NM_203371.1	11
2.4	−1.8	C1S	complement component 1, s subcomponent	NG_011694.1	12
9.7	−2.4/+2.5	DCN	decorin	NG_011672.1	12
6.1	4.1	MFAP5	microfibrillar associated protein 5	NM_003480.2	12
2.6	−2	NTN4	netrin 4	NM_021229.3	12
−6.6	−2.3	SLC38A1	solute carrier family 38, member 1	NM_001077484	12
−6.6	2.5	KPNA3	karyopherin alpha 3 (importin alpha 4)	NM_002267.3	13
4.8	2.8	LMO7	LIM domain 7	NM_005358.5	13
37.9	−4.6	POSTN	periostin, osteoblast specific factor	NM_001135934	13
3.9	3.4	CHURC1	churchill domain containing 1	NM_145165.2	14
4	11.7	EIF2S1	eukaryotic translation initiation factor 2, subunit 1 alpha, 35 kDa	NM_004094.4	14
−12.5	−2.7	IGF1R	insulin-like growth factor 1 receptor	NG_009492.1	15
4.5	19	FLJ37644	hypothetical LOC400618	NC_000017.10	17
2.6	13.3	ID1	inhibitor of DNA binding 1, dominant negative helix-loop-helix protein	NM_002165.2	20
4.8	−2.4	BEX1	brain expressed, X-linked 1	NM_018476.3	X

### Genomic clusters of misexpressed genes in the *LMNA* mutant heart

**Figure 2:**
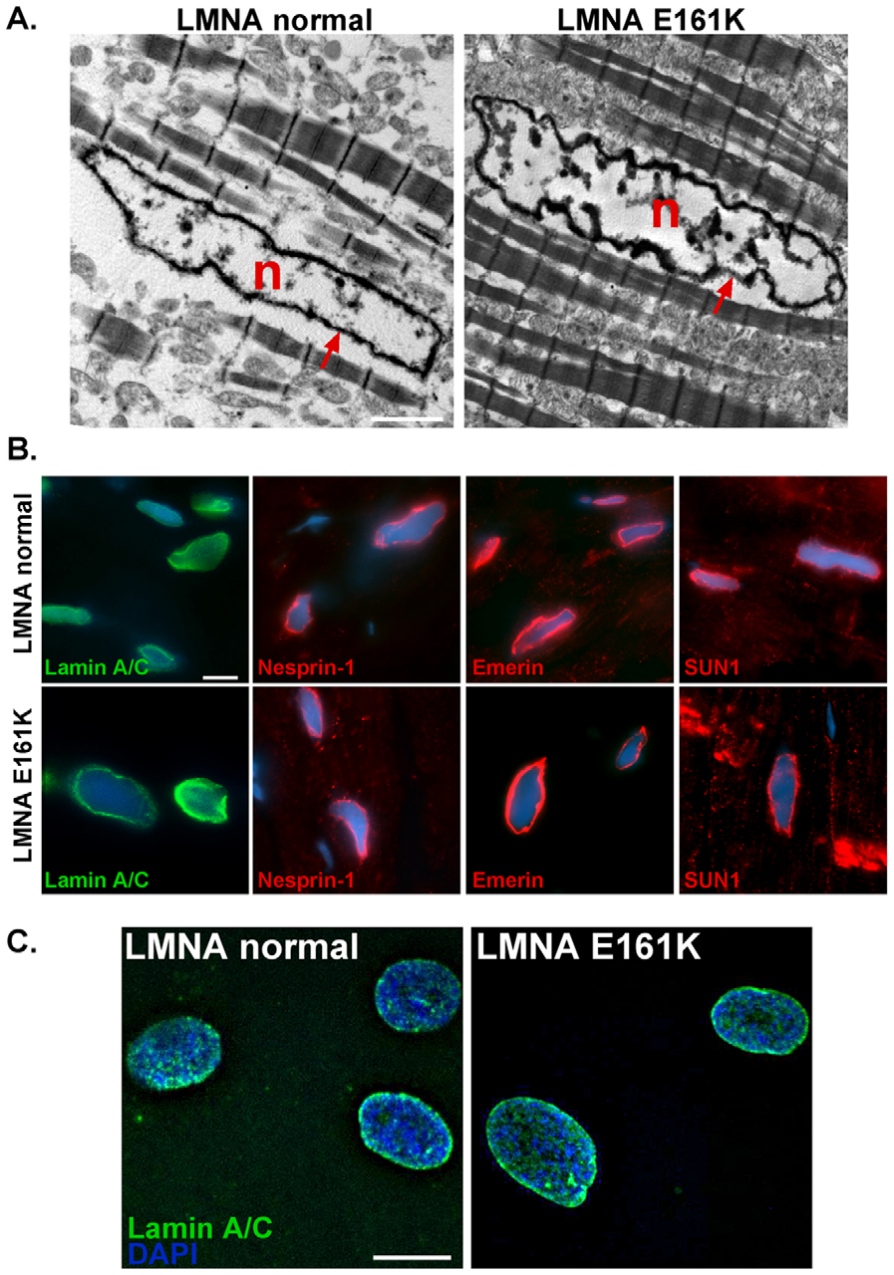
The lamina is intact in *LMNA* E161K heart and fibroblasts. (A) Electron microscopy illustrates the electron dense lamina in both the *LMNA* E161K and *LMNA* normal hearts, and shows no appreciable difference. N = nucleus, red arrows indicate nuclear membrane. Scale bar  = 2 µm. (B) The LINC complex proteins localize normally in *LMNA* E161K mutant heart. Sections from *LMNA* E161K mutant and *LMNA* normal heart were analyzed by immunofluorescence microscopy using antibodies for lamin A/C (green), nesprin-1, emerin and SUN1 (red). DAPI is shown in blue. Scale bar  = 10 µm. (C) Lamin A/C (green) localization was determined using immunofluorescence microscopy in *LMNA* E161K mutant and *LMNA* normal fibroblasts. DAPI shown in blue. Scale bar  = 10 µm.

Altered nuclear architecture and disorganized chromatin have been described with *LMNA* mutations [20], [27], [28]. Electron microscopy was used to examine the same *LMNA* mutant and *LMNA* normal cardiomyopathic hearts used for gene expression profiling. Abnormalities of nuclear shape were readily detected in the *LMNA* mutant but not the *LMNA* normal heart (Figure 2). The electron dense layer of heterochromatin immediately interior to the inner nuclear membrane appeared intact in the *LMNA* mutant heart and was not significantly different in density (n = 15 nuclei). The LINC complex, which links the nucleus to the cytoplasm, includes lamin A/C, the nesprins and SUN proteins [29]. We found that these components were normally localized in the *LMNA* mutant heart (Figure 2B). We also investigated lamin A/C in the *LMNA* E161K mutant fibroblasts and found normal localization (Figure 2C). Together these data support a model where protein components are localized to the nuclear membrane but where we anticipate that they function abnormally similar to what we previously noted with nesprin-1 mutations [30], [31].

A normally localized but abnormally assembled nuclear lamina may lead to disrupted or enhanced chromatin contacts and, therefore, abnormal gene expression. In this model, we would expect that contiguous regions of DNA, containing one or more genes, may be misregulated. To examine this possibility, we used the microarray data from the *LMNA* E161K heart, as these misexpressed genes may be relevant to disease pathogenesis. We queried whether the misexpressed genes in the *LMNA* E161K mutant heart shared genomic positioning. We examined the 583 genes identified in the microarray analysis for evidence of genomic clustering by using a sliding window analysis of gene density. This process identified regions of the genome where adjacent misregulated genes accounted for 75% or greater of the gene density in the region. Ten regions containing pairs of misexpressed genes were identified, and these misexpressed clusters are listed in Table 2. To determine the significance of identifying ten clusters with such an analysis, we performed 1000 simulations without obtaining a similar result, giving an empirical P value of <0.001.

**Table 2:** Genomic clusters of misexpressed genes.

Chromo-some	Gene Symbol	Fold Change (Mutant: Control)	Gene Symbol	Fold Change (Mutant: Control)	qPCR validation
2	CRIM1	−3.7	FEZ2	−4.2	
3	BBX	−3.8	CD47	3.4	
5	CSPG2	7.2	EDIL3	4	
8	ASAH1	2.4	PSD3	−9.5	
10	ITGB1	3.3	NRP1	−2.5	
12	WNK1	−7.5	RAB6IP2	−5.2	
12	LUM	3.3	DCN	9.4	
13	LMO7	−4.8	KCTD12	−4.4	−9.3, −4.6
13	MBNL2	−4.2	RAP2A	−3.8	−10.3, −1.4
16	LOC388279	3.1	MMP2	2.8	

Shown are genes that are misexpressed in the LMNA mutant heart that are colocalized in the same genomic interval. The chromosome position is shown on the left. The two genes within each interval are indicated in the subsequent columns.

### Two gene clusters on chromosome 13 are both centrally displaced in *LMNA* E161K mutant nuclei

From this list of ten misexpressed gene clusters (Table 2), we selected two of these clusters for further analysis (13A and 13B). Both clusters are found on chromosome 13, and each cluster harbors genes linked to striated muscle dysfunction. The 13A cluster contains *LMO7* and *KCTD12,* and the 13B cluster contains *MBNL2 and RAP2A.* LMO7 interacts with and regulates emerin, a nuclear membrane protein that is defective in X-linked Emery Dreifuss Muscular Dystrophy [10], [32]. The second gene cluster contains *MBNL2*, a member of the muscleblind family that contributes to the missplicing in myotonic muscular dystrophy [33], [34], [35]. Both Emery Dreifuss Muscular Dystrophy and myotonic dystrophy have significant cardiac involvement that preferentially affects the cardiac conduction system. In order to validate the expression results from the microarray analysis, quantitative real time PCR (qPCR) of independent cDNA preparations from the *LMNA* E161K mutant and *LMNA* normal hearts was conducted for the four genes in the two genomic clusters. Three of the four, *MBNL2*, *LMO7*, and *KCTD12* showed expression levels by qPCR that were in the same direction and of similar magnitude as those found in the microarray experiment, with decreased expression in the *LMNA* E161K mutant heart relative to the *LMNA* normal cardiomyopathic heart. The fourth gene, RAP2A, showed a modest decrease in expression in the *LMNA* E161K mutant heart relative to the *LMNA* normal cardiomyopathic heart, but to a lesser magnitude than the microarray results.

**Figure 3:**
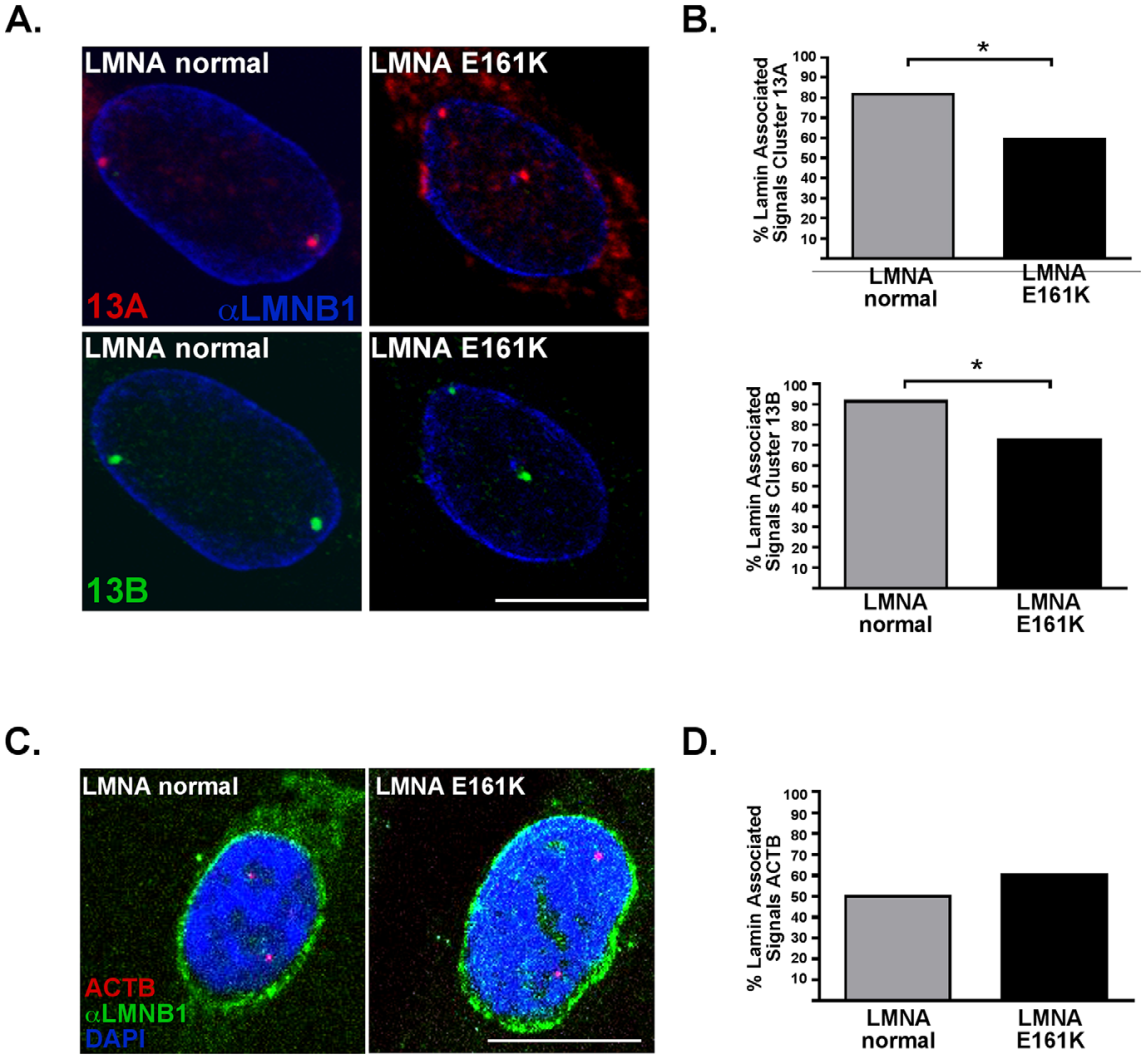
Two gene clusters on chromosome 13 are displaced from the nuclear periphery in *LMNA* E161K cells. Gene expression profiling identified gene clusters that were misexpressed in *LMNA* E161K. Two clusters from chromosome 13, referred to as 13A and 13B were studied because they contain genes important for striated muscle function. (A) Cluster 13A contains *LMO7* which encodes a nuclear membrane associated emerin-interacting protein. Cluster 13B contains *MBNL2.* The intranuclear position of Cluster 13A (red, top) and Cluster 13B (green, bottom) is shown in *LMNA*-normal nuclei and *LMNA*-mutant nuclei. Anti-Lamin B-1 (αLMNB1) is shown in blue. (B) The nuclear position of Cluster A was displaced away from the nuclear periphery in *LMNA* mutant versus normal (n = 98 control nuclei and n = 64 E161K nuclei), (*p = 0.0001)(top). Similarly, the nuclear position of Cluster B was displaced towards the nuclear center in *LMNA* E161K mutant versus *LMNA* normal nuclei (n = 106 control nuclei and n = 66 for E161K nuclei) (*p = 0.02) (bottom). (C) The nuclear position of the *ACTB* gene encoding β-actin was examined as a control genomic locus and did not differ between mutant and normal. Anti-Lamin B-1 (αLMNB1) is shown in green and DAPI staining in blue. Scale bar = 10 µm. (D) There was no significant difference between the localization of the ACTB locus in E161K *LMNA* mutant versus *LMNA* normal nuclei.

We next sought to determine whether the genomic regions with misexpressed genes also displayed aberrant intranuclear positioning. Because the quality of the explanted heart tissue did not permit us to conduct the study using cardiac material, we used fibroblasts from the same E161K individual as a surrogate. Cluster 13A contains LMO7 which is misexpressed in both *LMNA* E161K heart and fibroblasts. We performed 3D FISH in *LMNA* E161K fibroblasts using BAC probes to each of the two clusters, 13A and 13B (Figure 3). *LMNA* E161K mutant nuclei (n = 64 nuclei) and *LMNA* normal nuclei (n = 98 nuclei) were analyzed for the nuclear localization of cluster 13A. Signals were scored based on physical continuity with the lamin B signal at the nuclear periphery and given a value of 0 or 1 for absence or presence of contact with the lamina respectively. Binary data was analyzed using a standard t-test. There was differential localization of this gene cluster within the nucleus with 81.6% of signals in contact with the lamina in *LMNA* normal nuclei while only 59.4% of cluster 13A FISH signals were in contact with the lamina in *LMNA* E161K mutant nuclei (Figure 3B, top) (p = 0.0001). Similarly, cluster 13B FISH signals were centrally displaced in the *LMNA* E161K mutant nuclei with 91.5% of FISH signals in contact with the lamina in the *LMNA* normal nuclei (n = 106 nuclei) and 72.7% of signals in contact with the lamina in *LMNA* E161K mutant nuclei (n = 66 nuclei) (Figure 3B, bottom) (p = 0.02). These data indicate a significant re-localization of gene clusters 13A and 13B toward the center of the nucleus in the *LMNA* E161K mutant nuclei compared to the *LMNA* normal nuclei.

As a control, we also analyzed the β-actin locus on human chromosome 7 (Figure 3C). Chromosome 7 did not contain any genomic clusters of misregulated genes and therefore, we did not expect the genomic localization of chromosome 7 loci to be altered compared to control fibroblasts. *LMNA* E161K mutant nuclei (n = 20 nuclei) and *LMNA* normal nuclei (n = 20 nuclei) were analyzed by 3D FISH for the nuclear localization of the *ACTB (*β-actin) probe. No differential localization of *ACTB* was observed. *LMNA* normal nuclei showed peripheral localization of the signal in 50% of cells, while *LMNA* E161K mutant fibroblasts showed peripheral localization of the signal in 60% of cells (Figure 3D) (p = 0.3751). These data indicate that mislocalization in the *LMNA* E161K is not a general phenomenon and instead supports a model where specific genes are mislocalized.

### Reduced compaction of chromosome 13 gene clusters in *LMNA* E161K mutant nuclei

**Figure 4:**
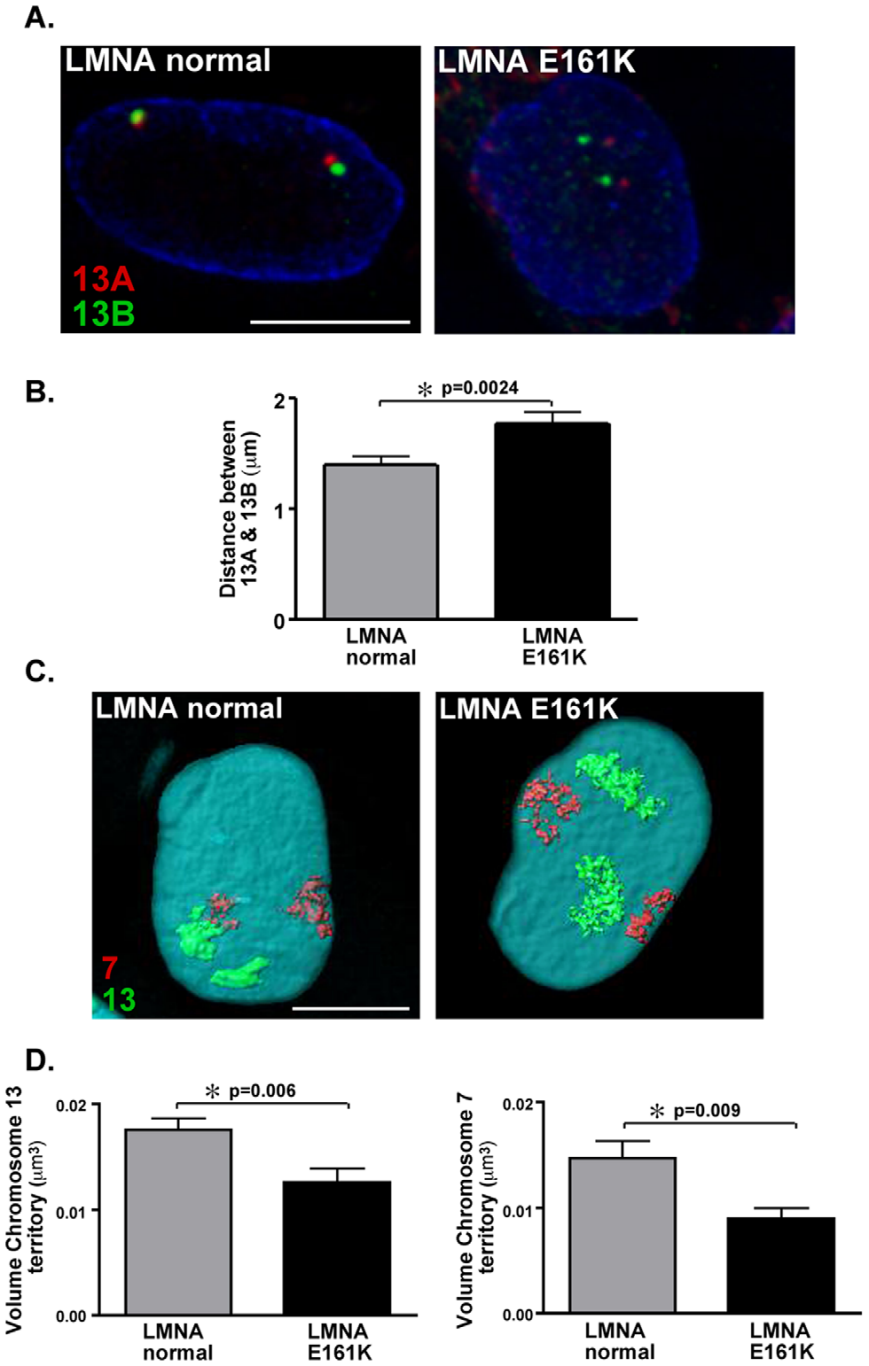
Increased distance between clusters 13A and 13B in *LMNA* E161K mutant nuclei. (A) The distance between Clusters 13A and 13B was measured in *LMNA* normal and *LMNA* E161K mutant nuclei (n = 56 and n = 94 respectively), 13A = red, 13B = green, anti-lamin B1 = blue. (B) The clusters are significantly further apart in the *LMNA* mutant nuclei than in the control nuclei consistent with a reduced compaction of the chromosome in this region, (*p = 0.0024). (C) The chromosome territory volume of chromosome 13 (green) and chromosome 7 (red) was reduced in *LMNA* E161K compared to *LMNA* normal fibroblasts. DAPI is blue. (D) Both chromosome 13 (left) and 7 (right) territories are significantly more compact in the *LMNA* E161K mutant fibroblasts. Scale bar = 10 µm.

We also found that the distance between gene clusters 13A and 13B differed between *LMNA* E161K mutant and *LMNA* normal nuclei. These two gene clusters span chromosome 13 from 13q22.2 to 13q34. Cluster 13A begins at position chr13:75,092,571 in the human genome, while cluster 13B begins at position chr13:96,672,575 (http:genome.ucsc.edu, Human May 2004 Assembly (hg17)). The two gene clusters are located 21 Mb apart in the genome, and therefore, a degree of proximity would be expected for the two FISH signals. However, this genomic distance was not consistent with the distances observed between the DNA FISH signals in the *LMNA* E161K fibroblasts. The mean distance between the two gene clusters in the *LMNA* E161K mutant nuclei (n = 56 nuclei) was 1.778±0.0957 µm, while the mean distance between the two gene clusters in the *LMNA* normal nuclei (n = 94 nuclei) was 1.401±0.0750 µm (Figure 4B) (p = 0.002). The significantly increased intersignal distance in the *LMNA* E161K mutant nuclei compared to the control indicates reduced compaction between cluster 13A and cluster 13B genes in *LMNA* E161K mutant cells.

We then analyzed the chromosome territories of both chromosome 13 and chromosome 7 to query whether chromosome compaction was globally altered in the *LMNA* mutant cells. Three-dimensional FISH of chromosome 13 and 7 was carried out in fibroblast cells using chromosome 13 or 7 paint as a probe and the chromosome territory volumes were calculated (Figure 4C). All territory volume measurements were normalized to nuclear volume. The volume of chromosome 13 was 0.017±0.001 µm^3^ in *LMNA* normal versus 0.013±0.001 µm^3^ in *LMNA* E161K fibroblasts (n = 27 and 19 nuclei respectively, p = 0.007)(Figure 4D, left). The chromosome 7 territory was 0.015±0.001 µm^3^ in *LMNA* normal versus 0.009±0.001 µm^3^ in *LMNA* E161K fibroblasts (n = 27 and 19 nuclei respectively, p = 0.009)(Figure 4D, right). We also compared nuclear volume in both *LMNA* normal and *LMNA* E161K fibroblasts and found no difference (586.4±37.7 n = 27 and 597.2±21.7 n = 19, respectively, p = 0.83). These data support that the overall volume of chromosome 13 and 7 was reduced in *LMNA* mutant cells. The observation of reduced compaction between the two intervals on chromosome 13, viewed in light of the overall reduction in chromosomal volume, suggests perturbed and uneven chromosomal architecture with the *LMNA* mutation.

### Chromosome 13 genes are more likely to be misexpressed in both *LMNA* E161K mutant heart and fibroblasts

**Figure 5:**
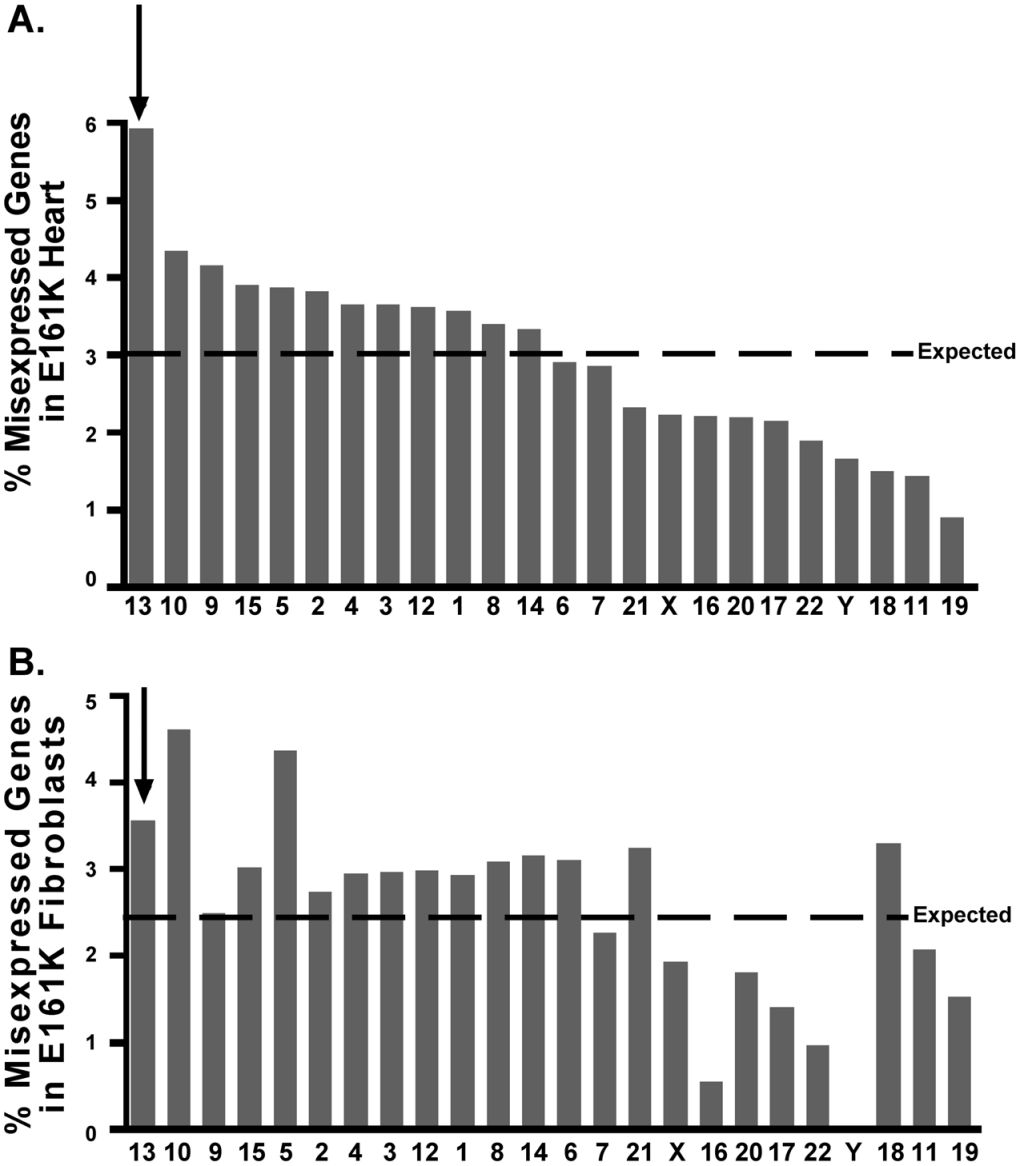
Chromosome 13 has a higher than expected percentage of misexpressed genes in both *LMNA* mutant heart and fibroblasts. Gene expression was compared between *LMNA* E161K mutant and *LMNA* normal hearts (A) or fibroblasts (B). Chromosomes are indicated along the x-axis. Percent misexpressed genes for entire genome is drawn as expected line. Percent misexpressed genes per chromosome is represented by gray bars. Arrows indicate chromosome 13.

The presence of two genomic clusters on chromosome 13 and the mislocalization of the two chromosome 13 clusters led us to investigate whether chromosome 13 itself was mislocalized in *LMNA* E161K mutant cells. Based on the gene expression analysis in the heart, approximately 3% of genes present on the chip were misexpressed in the *LMNA* E161K mutant heart (583 misexpressed genes out of 18350 total genes). We therefore expect that if the misexpressed genes are randomly distributed in the genome approximately 3% of genes on each chromosome will be misexpressed. In Figure 5A, the percentage of misexpressed genes on each chromosome is shown. Chi-square analysis indicates that the misexpressed genes are not randomly distributed among the chromosomes and that some chromosomes are more affected by the *LMNA* E161K mutation than others (χ^2^ = 39.7, df = 23, p = 0.016). Chromosome 13 had the highest percentage of misexpressed genes at 6% (20 misexpressed genes out of 340 genes representing chromosome 13 on the chip). The percentage of misexpressed genes did not correlate with chromosome size or gene density (data not shown).

We also interrogated gene expression changes from *LMNA* E161K mutant fibroblasts. Analysis in the fibroblasts revealed that approximately 2.5% of the genes present on the chip were misexpressed in the *LMNA* E161K mutant fibroblasts (468 misexpressed genes out of 18350 total genes). Using the same analysis as in the heart, we expect 2.5% of genes on each chromosome to be misexpressed if the genes are randomly distributed. We found that in the *LMNA* E161K mutant fibroblasts, as in the *LMNA* E161K mutant heart, genes were not misexpressed randomly among the chromosomes (χ^2^ = 37.5, df = 23, p = 0.029)(Figure 5B). The chromosomes most likely to show gene expression changes in the *LMNA* E161K mutant fibroblasts closely reflect the chromosomes most affected in the *LMNA* E161K mutant heart, including chromosome 13. It is also notable that genes misexpressed in both the *LMNA* E161K heart and fibroblasts are over-represented on chromosome 13. We found that 3 of the 36 shared genes misregulated in both *LMNA* E161K heart and fibroblasts are found on chromosome 13. These genes represent 0.9% of chromosome 13 genes represented on the microarray chip. We validated the misexpression of several of the genes on chromosome 13 in the fibroblasts using qPCR. Three of the four genes we analyzed were misregulated in the *LMNA* E161K mutant fibroblasts in the same direction as on the microarray. CKAP2, LMO7 and KPNA3 were upregulated and PCDH9 was upregulated in the qPCR, but down regulated in the microarray. The next highest chromosome demonstrating misexpression was chromosome 12 with only 0.5% of its genes misregulated in both *LMNA* E161K heart and fibroblasts. These data indicate that chromosome 13 may be preferentially affected by this *LMNA* E161K mutation.

### Chromosome 13 is mislocalized in the nuclei of *LMNA* mutant fibroblasts

**Figure 6:**
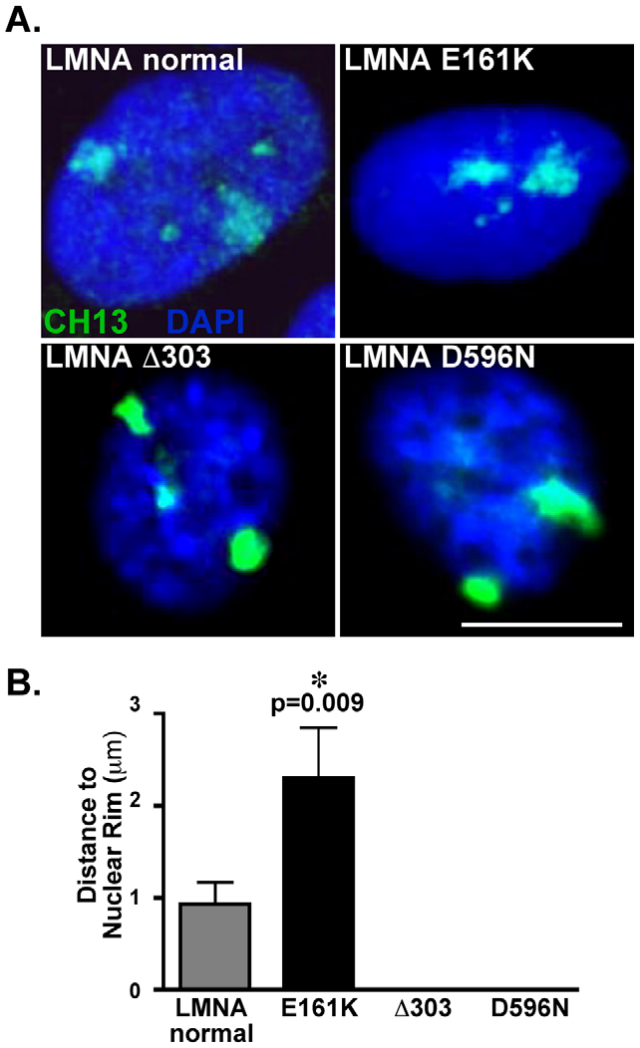
The chromosome 13 territory is displaced in *LMNA* mutant nuclei. (A) Nuclear position of the chromosome 13 territory was determined by chromosomal painting, shown as green. The position of chromosome 13 is shown for *LMNA* normal and mutant nuclei. DAPI staining (blue). (B) Chromosome 13 territories are significantly displaced in the *LMNA* mutant nuclei compared to the control nuclei (n = 30 control nuclei and n = 15 *LMNA* E161K (p = 0.009), n = 12 Δ303, n = 10 D596N).

To determine if the abundance of misexpressed genes found on chromosome 13 may be a consequence of changes in chromosome 13′s interaction with the lamina, we analyzed the chromosome territory of chromosome 13. Three-dimensional FISH of chromosome 13 was carried out in fibroblast cells using chromosome13 paint as a probe. *LMNA* E161K mutant nuclei (n = 15 nuclei) and *LMNA* normal nuclei (n = 30 nuclei) were analyzed for the nuclear localization of chromosome 13 (Figure 6A). Localization was determined by measuring the minimum distance from the edge of a chromosome territory to the edge of the DAPI staining in the x, y, and z planes. Differential localization of the chromosome 13 territory within *LMNA* E161K mutant nuclei was observed, with an average minimum distance between the edge of a chromosome 13 territory and the edge of the DAPI staining of 2.0±0.5 µm in the *LMNA* E161K mutant nuclei, and 0.9±0.2 µm in control nuclei (Figure 6B), (p = 0.0088). Notably, the internal displacement of the entire chromosome 13 is consistent with what was seen for the individual chromosome 13 clusters.

To determine if chromosome 13 is mislocalized in other *LMNA* mutant fibroblasts, we evaluated the intranuclear position of chromosome 13 in two different fibroblast cell lines, each derived from patients with striated muscle disease. *LMNA* Δ303 or *LMNA* D596N each independently are known to cause dominantly inherited familial cardiomyopathy [36], [37]. 3D-FISH analysis revealed that the position of chromosome 13 within the nuclei of both the *LMNA* Δ303 and *LMNA* D596N mutant fibroblasts was grossly abnormal. In both *LMNA* mutants, chromosome 13 was in direct contact with the DAPI edge; the minimum distance between the edge of a chromosome 13 territory and the edge of the DAPI staining was 0 µm for both Δ303 and D596N, (n = 12 and 10 nuclei, respectively) compared to 0.9±0.2 µm in *LMNA* normal nuclei (Figure 6B). These data suggest that chromosome 13 mislocalization, in either direction, may be an important aspect of *LMNA* mutations.

To evaluate the proliferative status of the fibroblast cell lines used, we analyzed Ki-67 to determine the level of senescence of each culture. Ki-67 antibodies react with a nucleolar antigen that is only present in proliferating cells providing a marker to estimate the fraction of a culture in a growth state [38]. Previous studies by Al-Baker and colleagues considered cultures with a Ki-67 index below 0.02 senescent and cultures above 0.4 “young” and proliferative [39]. We performed Ki-67 indexes at the time of harvest for FISH analysis. Each cell line had approximately 40% or greater levels of Ki-67 positive nuclei (*LMNA* normal 88%, E161K 66%, Δ303 47% and D596N 77% n = 100 nuclei per culture). We also examined the localization of the chromosome 13 territory in Ki-67 positive and Ki-67 negative cells in the *LMNA* normal fibroblasts. The distance of the chromosome 13 territory to the nuclear rim was not affected by Ki-67 status (0.794±0.162, n = 34 and 0.524±0.203, n = 21, Ki-67 positive and negative, respectively, p = 0.30). These data indicate that proliferative status was not correlated with the abnormal localization of chromosome 13 in the fibroblast cultures.

## Discussion

Mutations in the nuclear membrane gene, *LMNA*, cause inherited human disease. Many *LMNA* mutations have been linked to striated muscle disease where there is progressive weakness of skeletal and cardiac muscle and often concomitant cardiac conduction system disease. Within *LMNA* mutant families, there are strikingly variable phenotypes and the mechanism by which an individual *LMNA* missense mutation causes disease may be mutation specific. In this study, we examined the gene expression changes and chromosomal positioning of misexpressed loci from a *LMNA* mutation, E161K. This mutation was previously associated with inherited cardiomyopathy [25], [26]. The distribution of *LMNA* mutations has challenged genotype-phenotype correlations since mutations associated with striated muscle disease can map anywhere within lamins A and C and may be associated with gain of function, dominant negative effects, loss of function, as well as haploinsufficiency [40]. Only the lipodystrophy and progeria phenotypes have shown some evidence of a genotype-phenotype relationship. We hypothesize that distinct mutations, even those mutations associated with a similar phenotype, may produce disease through unique and differential effects on chromosome positioning and gene expression.

We analyzed heart from a single *LMNA* mutation, E161K. We found that while nuclear architecture was disturbed, the appearance and positioning of the heterochromatin and protein components of the LINC complex, including lamin A/C, were intact. From this, we expect that the inner nuclear membrane complex is normally localized but abnormally assembled at its site. Incorporation of mutant lamin A/C into the nuclear lamina may provide an uneven surface that fails to normally interact with chromatin. The precise chromosomal markings for lamin A/C interaction have yet to be defined, but our data would suggest that chromosome 13 may harbor sequences more likely to be altered by a mutant nuclear lamina. It is interesting to note that chromosome 13 is among the most gene poor chromosome with a high percentage of noncoding region [41].

**Figure 7:**
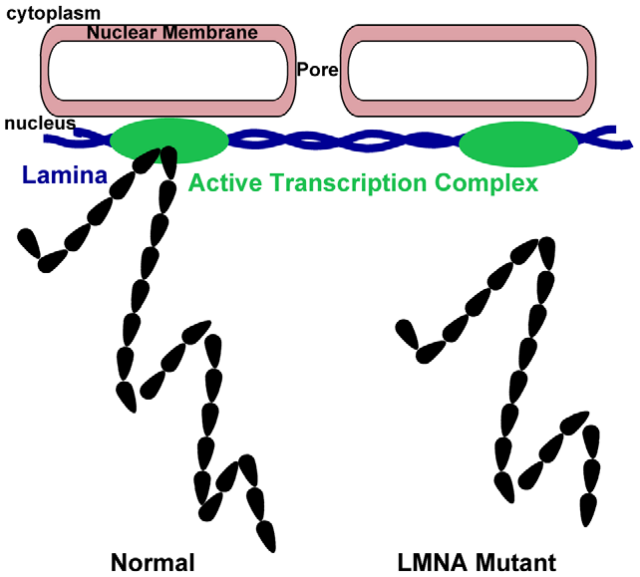
Model of chromatin positioning and gene expression. In the case of the *LMNA* E161K mutation, two distinct loci on chromosome 13 were displaced to a more intranuclear position (right). We hypothesize that loss of interaction with the lamina (blue) prevents interaction with active chromatin complexes (black) and reduces gene expression.

Gene expression profiling of *LMNA* E161K heart revealed genomic clustering of misexpressed genes. We examined two specific clusters located on chromosome 13 that contained genes relevant to striated muscle dysfunction. These two gene clusters on chromosome 13 (13A and 13B) showed significant changes in nuclear localization and were displaced toward the nuclear center, indicating a loss of contact with the nuclear lamina and redirection to more internal areas of the nucleus. This is consistent with a model where this *LMNA* mutation may be associated with partial release of the chromatin from the nuclear periphery (Figure 7). Curiously, central displacement was associated with down-regulation of gene expression, arguing for transcriptional upregulation closer to the periphery and/or repression associated with central displacement. Although more commonly the nuclear periphery has been implicated in gene repression (for review see [42]), transcriptional complexes have also been localized to the nuclear periphery. Specifically, active genes have been shown to associate with the nuclear pore complex (reviewed in [43]). In yeast the transcription of multiple genes, including *GAL* and *HXK1*, has been shown to be localized to the nuclear pore upon activation [44], [45], [46]. In *Drosophila,* the SAGA histone acetyl transferase complex has been implicated in localizing heat-shock loci to the nuclear pore and enhancing transcription [47]. The MSL complex in Drosophila is involved in dosage compensation of the male X chromosome, resulting in a 2-fold upregulation of genes. The MSL complex has also been shown to interact with nuclear pores [48]. In mammalian cells, it has been shown that transcriptional complexes can be associated at the nuclear periphery but that an interaction between lamin B and lamin A/C is required for normal regulation [17].

We also found evidence for abnormal chromatin compaction of the chromosome 13 gene clusters. The region of chromosome 13 containing the two clusters was less compact in the *LMNA* E161K mutant fibroblasts, but the chromosome 13 volume was smaller. The smaller chromosome volume could reflect the distinctly abnormal nuclear shapes that are well described in *LMNA* mutations and that were also seen here. We favor that reduced chromosome volume is not linked to gene expression since chromosome 7 also showed a reduction in volume. The looser chromatin configuration seen proximal to the misexpressed gene clusters may be an effect of abnormal gene expression. The greater distance between these two clusters in *LMNA* mutant nuclei is consistent with a more relaxed and open conformation to the chromatin in this genomic region and may also reflect altered interactions with transcription factors and transcriptional machinery. This reduced compaction may correlate with increased gene expression as LMO7, one of the genes in cluster 13A is upregulated in the mutant fibroblasts.

We found that chromosome 13 was more likely to have misexpressed genes than other chromosomes and we also found that chromosome 13 was displaced in all *LMNA* mutants examined. A prior study examined chromosome territories in response to *LMNA* mutations and found that chromosomes 13 and 18 were displaced from the periphery to the interior in some *LMNA* mutant cell lines [19]. Our independent data confirms central mislocalization of chromosome 13 in *LMNA* E161K mutant nuclei and now correlates the finding with aberrant gene expression of chromosome 13-associated loci. Meaburn and colleagues also noted an intermediate localization of chromosome 13 in two *LMNA* mutant fibroblast lines, consistent with the notion that different mutations may affect chromosome 13 localization differently [19].

The role of the nuclear membrane as an active regulator of gene expression and other nuclear functions has been increasingly appreciated. Therefore, it follows that in cases of an abnormally assembled nuclear membrane infrastructure, such as that with *LMNA* gene mutations, there is abnormal nuclear positioning, chromosome compaction and gene expression. The number of individual *LMNA* gene mutations now exceeds more than 300, and it is possible that the unique conformation resulting from each of these mutations may be associated with distinctly abnormal nuclear architecture. Alternatively, there may be specific malpositioning of intranuclear chromosome position linked to certain disease phenotypes. The data herein suggest that chromosome 13 may be particularly relevant to cardiomyopathy and cardiac conduction system disease.

## Materials and Methods

### Tissue Samples and Culture

An adult male patient with the *LMNA* E161K gene mutation underwent a heart transplant at the University of Chicago, and tissue was harvested from the left ventricle of the explanted heart. Fibroblast cell lines were derived from two other *LMNA* mutant patients with *LMNA* Δ303 or *LMNA* D596N mutation. As a control for the heart microarray studies, explanted heart tissue was isolated from an adult male receiving a heart transplant for ischemic dilated cardiomyopathy. Two normal control fibroblast cell lines were obtained from American Type Culture Collection (ATCC), cell line CRL-2565 and 06.1311. Written and informed consent from all human subjects was obtained in accordance with the University of Chicago's Institutional Review Board. All work was done under the approval of the University of Chicago Institutional Review Board. Evaluations performed at the University of Chicago Cardiology Clinics were used to obtain clinical data. Family medical records were obtained after the patient contacted family members. Family history was obtained from the patient by a certified genetic counselor.

### Immunofluorescence Microscopy

Heart sections from *LMNA* normal and *LMNA* E161K patients were fixed in ice-cold methanol for 2 min, rinsed in PBS and blocked with 5% FBS in PBS with 0.1%Triton for 1 hour at room temperature. Primary antibodies used and their concentrations were: AN1 1∶100 [49]; anti-Lamin A/C (Santa Cruz, cat. # SC 20681) 1∶30; anti-SUN1 1∶1000; anti-emerin (Novocastra, NCL-emerin) 1∶250. Sections were incubated with primary antibody in blocking buffer at 4°C overnight. Secondary antibodies conjugated to either Cy3 or Alexa 488 were incubated at room-temperature for 1 hour. The section were washed and mounted in Vectashield with 4′,6-diamidimo-2-phenylindole (DAPI) (Vector Labs), and images were captured with an Axiophot microscope and Axiovision (Carl Zeiss) software. Fibroblasts from *LMNA* normal and *LMNA* E161K patients were grown on slides, then fixed in ice-cold methanol for two minutes, rinsed in PBS and blocked with 5% FBS in PBS with 0.1%Triton for 1 hour at room temperature. Primary antibody used was α-human- Ki-67 (DAKO, MIB-1) at 1∶50 or anti-Lamin A/C (Santa Cruz, cat. # SC 20681) at 1∶30. Territory distances were measured without regard to Ki-67 status.

### Electron Microscopy

Tissue that had been formalin fixed and paraffin embedded was used for the ultrastructural studies. After removal with a razor blade the tissue was washed in xylene, 100% alcohol, 95% alcohol and 70% alcohol before transfer into 0.1M Milonig's buffer. After post-fixation in 1.0% osmium tetroxide the tissue was Epon embedded. Semithin (0.5 µm) toluidine blue stained sections were used to pre-screen the tissue and select areas for subsequent thin sectioning and electron microscopy (Philips CM10).

### Preparation of cDNA and Affymetrix Gene Chip Hybridization

Gene expression analysis was carried out by the Functional Genomics Facility at the University of Chicago according to the Affymetrix Expression Analysis Technical Manual [50]. Briefly, total RNA from left ventricle cardiac tissue and fibroblast cell cultures were extracted using Trizol Reagent (Invitrogen). cDNA was synthesized from 10 µg of total RNA using the Superscript Choice System (Invitrogen) and labeled using the BioArray High Yield RNA Transcript Labeling kit (Enzo Diagnostics). cDNA was hybridized to the Affymetrix Human Genome U133 Plus 2.0 Array. Microarrays were washed, stained, and scanned according to standard procedures.

### Microarray Data Analysis

Robust Microarray Analysis **(**RMA) and Affymetrix Microarray Suite 5.0 (Mas5) background corrections and normalizations were applied independently to the raw data sets using the Bioconductor open source software [51]. Differentially expressed genes for each background correction method were identified using RankProducts (RP) to rank the probesets by fold change [52]. The top 1000 differentially overexpressed probesets and the top 1000 differentially underexpressed probesets for each background correction were selected. The intersection of the two lists of overexpressed genes (RMA/RP and MAS5/RP) and the intersection of the two lists of underexpressed genes formed the resulting list of differentially expressed genes.

The list of differentially expressed genes was examined for genomic clustering using methods and programs previously published [53]. The list of differentially expressed genes and the list of genes covered on the Affymetrix Human Genome U133 Plus 2.0 chip were analyzed with a 500 Kb sliding window with a 100 Kb slide. Windows of interest were selected if the gene density of the differentially expressed genes was 75% or greater than the gene density of the genes on the Affymetrix chip for that region. Regions where the gene density exceeded the 75% threshold and that contained more than one differentially expressed gene defined a genomic cluster.

To assess the significance of the number of genomic clusters identified in our dataset of differentially expressed genes, we employed a simulation strategy. The above scripts from Bisognin et al. were used by an additional script to calculate the number of genomic clusters for 1000 randomly generated lists of 675 probesets [53]. The mean number of genomic clusters was 3.4, with a range of 0 to 9 clusters.

All data has been deposited in a MIAME compliant database. Accession numbers for the GEO database are as follows: GSM435885, GSM435886, GSM435887.

### qPCR Validation of Microarray Results

Gene expression fold changes in the heart and fibroblasts were validated using quantitative polymerase chain reaction (qPCR) of cDNA. Reactions were carried out in triplicate, with three independent replicates, according to manufacturer's directions using SYBR GreenER (Invitrogen). Primers for the heart analysis were as follows: GAPDH F: TCGACAGTCAGCCGCATCTTCTTT; GAPDH R: ACCAAATCCGTTGACTCCGACCTT; KCTD12 F: CAGCAAACGTTGACTTCTGGGCAA; KCTD12 R: ATCTACAGATAGGCAGCCCTTGGT; LMO7 F: TTGCTCCTCCAAGGCACCATAAGA; LMO7 R: AGAGGAGCAGCTTGTCAATGACCT; MBNL2 F: ATCACCATGGCTTTGAACGTTGCC; MBNL2 R: TCATCAGAGCGTGAGCATGTTCCT; RAP2A F: AGGGAAGCAACTGTGATGGGAAGA; RAP2A R: GTGAGGTTTCTGCAAACGGGAACA.

Primers for the fibroblast analysis were as follows: CKAP2 F: TTGACCAGCGAAGACATACTG; CKAP2 R: TCTTCCTTTGCCAGCTTTCC; LMO7 F: TGTTGCCTGTGAGTGTGAC; LMO7 R: ACAGTGCTTTCGTATGGAGG; KPNA3 F: TTTCTTGTGCCCCTTCTGAG; KPNA3 R: GTGTGATAAGAGATTTGGGAAGTG.

### 3D Immuno-FISH

3D Immuno-FISH was performed according to [54]. Bacterial artificial chromosome (BAC) DNA for FISH probes was isolated by standard alkali lysis protocols. BACs were labeled with digoxigenin dUTP or biotin dUTP using a Nick Translation Kit (Roche Applied Science). The *ACTB* gene was detected using BAC RP11-754B14. The *LMO7/KCTD12* gene cluster was detected using BAC RP11-587B15 labeled with digoxigenin. The *MBNL2/RAP2A* gene cluster was detected using RP11-128N14 labeled with biotin. The chromosome 13 territory was detected using a FITC labeled human chromosome 13 paint (Cytocell).

The anti-Lamin B (M-20) antibody (Santa Cruz Biotechnology, Inc.) was used to detect Lamin B. Goat anti-rabbit Cy5 (Jackson ImmunoResearech Laboratories, Inc.) was used to detect the anti-Lamin B antibody. Anti-dig Cy3 (Jackson ImmunoResearch Laboratories, Inc.) and streptavidin FITC were used to detect the BAC DNA probes.

### Image Analysis

#### 3D Immuno-FISH Images

Images of patient and control nuclei were collected using the Olympus FluoView 1000 confocal fluorescent microscope. Optical sections were taken 0.12 microns apart through the entire nucleus for all images. This technique generated a stack of images that could be merged for a three dimensional view of the nuclei. FISH signals were scored as peripheral (in contact with the nuclear lamina) or central (no contact with the nuclear lamina) using ImageJ software. The View5D (http://www.nanoimaging.de/View5D/), Image5D (http://rsbweb.nih.gov/ij/plugins/image5d.html), RGB Stack Merge (http://rsbweb.nih.gov/ij/plugins/rgb-merge.html) and Stack Splitter (http://rsbweb.nih.gov/ij/plugins/splitter.html) plug-ins were utilized for this analysis.

The distance measurements between the *LMO7/KCTD12* and *MBNL2/RAP2A* gene clusters on the same homologous chromosome in each nuclei and the distance between alleles of the *LUM/DCN* gene cluster were obtained using the ImageJ plug-in SyncMeasure3D (http://rsb.info.nih.gov/ij/plugins/sync-windows.html). This plug-in performs measurements on three-dimensional regions of interest (ROI) in all selected and synchronized windows. Using the ROI manager, the x, y, and z coordinates of each ROI (each FISH signal) are measured in µm. The distance between two ROIs was obtained using the formula (x^2^+y^2^+z^2^)^1/2^ µm.

The distance from the edge of a chromosome territory to the edge of the nucleus was calculated using a macro in ImageJ. Prior to image analysis, all images were deconvolved using the Huygens software package (Scientific Volume Imaging, Hilversum, The Netherlands). Subsequent image processing and distance mapping were done with a macro written for ImageJ (National Institutes of Health, Bethesda, MD). All chromosome territory images were subjected to a despeckle (3×3 hybrid median filter) followed by thresholding and creation of a filled (no holes) binary image mask. The territory mask was then mapped onto a Euclidean Distance map of the DAPI signal for each slice. The minimum pixel intensity value of the DAPI EDM enclosed by the chromosome territory mask was recorded for each slice. The lowest pixel value was taken as the shortest distance from the edge of the chromosome territory to the edge of the nucleus in x-y. The image stack was then re-sliced perpendicular to the x-y axis and the process was repeated for each x-z slice. The minimum distance to the edge of the DAPI signal was again calculated for each particle. The minimum value for distance for the x-y or x-z axes was scored as the shortest distance from the edge of a territory to the edge of the nucleus for each chromosome territory in a nucleus. Volumes of chromosome territories and total nuclear volumes were calculated using surfaces feature of Imaris Software version 7.1.1 (Bitplane St. Paul, MN).

### Statistical Analysis

All data was analyzed using a student's t-test, standard deviation is provided in the text.
